# Consciousness and complexity: a consilience of evidence

**DOI:** 10.1093/nc/niab023

**Published:** 2021-08-30

**Authors:** Simone Sarasso, Adenauer Girardi Casali, Silvia Casarotto, Mario Rosanova, Corrado Sinigaglia, Marcello Massimini

**Affiliations:** Department of Biomedical and Clinical Sciences ‘L. Sacco’, University of Milan, Milan 20157, Italy; Instituto de Ciência e Tecnologia, Universidade Federal de São Paulo, Sao Jose dos Campos, 12247-014, Brazil; Department of Biomedical and Clinical Sciences ‘L. Sacco’, University of Milan, Milan 20157, Italy; IRCCS Fondazione Don Carlo Gnocchi ONLUS, Milan 20148, Italy; Department of Biomedical and Clinical Sciences ‘L. Sacco’, University of Milan, Milan 20157, Italy; Department of Philosophy, University of Milan, Milan 20122, Italy; Department of Biomedical and Clinical Sciences ‘L. Sacco’, University of Milan, Milan 20157, Italy; IRCCS Fondazione Don Carlo Gnocchi ONLUS, Milan 20148, Italy

**Keywords:** sleep, coma, anesthesia, information, integration

## Abstract

Over the last years, a surge of empirical studies converged on complexity-related measures as reliable markers of consciousness across many different conditions, such as sleep, anesthesia, hallucinatory states, coma, and related disorders. Most of these measures were independently proposed by researchers endorsing disparate frameworks and employing different methods and techniques. Since this body of evidence has not been systematically reviewed and coherently organized so far, this positive trend has remained somewhat below the radar. The aim of this paper is to make this consilience of evidence in the science of consciousness explicit. We start with a systematic assessment of the growing literature on complexity-related measures and identify their common denominator, tracing it back to core theoretical principles and predictions put forward more than 20 years ago. In doing this, we highlight a consistent trajectory spanning two decades of consciousness research and provide a provisional taxonomy of the present literature. Finally, we consider all of the above as a positive ground to approach new questions and devise future experiments that may help consolidate and further develop a promising field where empirical research on consciousness appears to have, so far, naturally converged.

## Introduction

Inferring the presence or the absence of consciousness from physical brain properties is a major challenge faced by scientists, with remarkable clinical and ethical implications. Decades of research on the physical substrate of consciousness did not ensure agreement on the topic, with theoretical proposals still diverging on fundamental assumptions and interpretations.

The aim of this review is to suggest that, despite this apparent disagreement, catching a glimpse of recent empirical data reveals a positive trend of convergence.

In the first section, we provide a systematic survey of the existing literature and document an emerging empirical consensus on a broad spectrum of complexity measures applied to brain signals as reliable indices of the presence/absence of consciousness across many different conditions, such as sleep, anesthesia, hallucinatory states, epilepsy, coma, and related disorders. In addition, we note that this host of studies were independently proposed by researchers employing different experimental approaches and endorsing disparate theoretical positions.

In the second section, we revisit early theoretical proposals linking consciousness to complexity. We show that the original principles were put forward more than 20 years ago with the definition of a specific form of complexity, arising from the coexistence of functional integration and differentiation in the brain. We then show that this rationale gave rise to novel measures and to original predictions.

In the third section, we argue that linking early principles and predictions to current complexity-related metrics can help interpret and conceptually organize the broad spectrum of current proposed methods and measures. Here, we highlight that they share a common denominator as they all tend to gauge the coexistence of functional integration and functional differentiation in the brain, albeit with different methods and assumptions. We thus provide a provisional taxonomy to organize existing complexity metrics and highlight their relationships and caveats.

In the last section, we use all the above as a solid foothold to approach new questions and devise future experiments. Among others, we consider the problems of understanding the neuronal mechanisms, the spatiotemporal grain, and the localization of complexity in the brain, as well as the difference between observational and perturbational approaches to estimating complexity. Finally, we argue that addressing these issues may help refine methods for assessing consciousness and foster constructive exchange among different theoretical frameworks on solid empirical grounds.

## Current empirical convergence

An outstanding problem in the empirical science of consciousness is identifying the fundamental physical brain properties that reliably index the presence and the absence of consciousness. Here, the presence of consciousness is operationalized as the presence of immediate reports (e.g. those obtained from subjects during wakefulness) and delayed reports (e.g. those obtained from subjects waking up from unresponsive states such as dreaming and ketamine anesthesia), as well as minimal but reliable behavioral signs (such as those provided by patients in a minimally conscious state). By contrast, the absence of consciousness is operationally defined as the absence of all of the above.

In this context, consciousness is defined as the capacity for any kind of experience, a concept that is upstream to further distinctions, such as those between levels (Laureys 2005; Boly *et al.* 2013), those between global states of consciousness (Bayne *et al.* 2016; Mckilliam 2020) (e.g. the distinction between dreaming and wakeful consciousness), and those between local states of consciousness (Bayne *et al.* 2016) characterized in terms of specific conscious contents or phenomenal character.

Such primary distinctions between conscious and unconscious conditions is the focus of a substantial body of literature, a fundamental concern for clinicians, and arguably an important first step for understanding the relationship between consciousness and physical brain properties.

In this section, we unveil the emergence of a new class of empirical measures that have recently shown a remarkable performance in indexing the presence/absence of consciousness across a variety of conditions.

### Pointing the radar on complexity measures

The existence of a large body of works on complexity measures has only been recently recognized (Arsiwalla and Verschure 2018) but has never been systematically reviewed nor taxonomized, thus remaining somewhat unnoticed. One way to appreciate the emerging consilience of evidence on such measures is to contrast them against the background of the disagreements and conflicting empirical results characterizing other empirical measures of consciousness. A first example is represented by markers of consciousness based on event-related potentials (ERPs). In this case, several studies (Bekinschtein *et al.* 2009; Faugeras *et al.* 2011, 2012) proposed a late, positive, fronto-parietal ERP evoked by visual or auditory stimuli (called P3b) as a reliable signature of consciousness. More recent studies (Pitts *et al.* 2014; Silverstein *et al.* 2015; Tzovara *et al.* 2015; Sergent *et al.* 2021), however, questioned this interpretation and opened a debate on the precise relationship of P3b to consciousness as opposed to task relevance, report, and decision processes that are associated with conscious access mechanisms. Most importantly in the present context, P3b was found to have a low sensitivity with respect to the presence of consciousness in clinical conditions (Faugeras *et al.* 2012; Sitt *et al.* 2014).

Another example of discrepancy is represented by indices based on global brain metabolic rates as they can be found decreased during loss of consciousness in deep sleep and anesthesia (Nofzinger *et al.* 2002; Kaisti *et al.* 2003), whereas they increase when consciousness is lost during generalized seizures (Bai *et al.* 2010). A similar problem applies to global spectral measures, such as alpha and delta electroencephalographic (EEG) power. Indeed, while the presence of a prominent alpha power typically provides a useful index of preserved brain function also in brain-injured patients, with the exception of postanoxic alpha-coma (Westmoreland *et al.* 1975), alpha is found consistently decreased in conscious conditions such as dreaming (Esposito *et al.* 2004), psychedelic-induced states (Timmermann *et al.* 2019), and in locked-in patients (Babiloni *et al.* 2010). On the other hand, while EEG delta power is typically found to negatively correlate with the presence of consciousness, important exceptions are found in the literature as EEG can show persistent large delta waves in conscious participants during the administration of the cholinergic antagonist atropine (Ostfeld *et al.* 1960) and of the gamma-aminobutyric acid (GABA) reuptake inhibitor tiagabine (Darmani *et al.* 2021) and during some instances of status epilepticus (Gökyiǧit and Çalişkan 1995; Vuilleumier *et al.* 2000), as well as in rare cases of children with Angelman syndrome (Sidorov *et al.* 2017; den Bakker *et al.* 2018; Frohlich *et al.* 2020). Notably, these and other dissociations have been systematically addressed by a very recent review article (Frohlich *et al.* 2021) further questioning the notion that high-amplitude delta oscillations recorded in humans at the scalp level are a reliable indicator of unconsciousness.

Against this backdrop, it becomes easier to recognize the emergence of a complementary profile in the relevant literature. Overall, a large body of work supports the notion that the presence of consciousness is invariably associated with high brain complexity, which, *vice*  *versa*, is found to be consistently decreased during physiological, pharmacological, or pathological-induced loss of consciousness.

**Figure 1.**
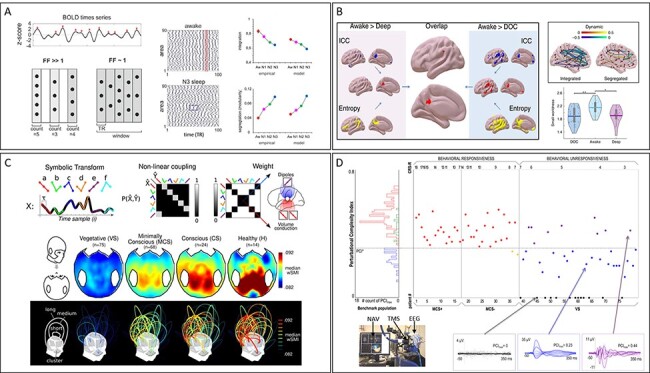
Relevant examples of brain complexity measures as a reliable index of the state of consciousness

Four examples of such measures, employing different experimental approaches, including both electrophysiology and functional brain imaging, and testing various conditions encompassing deep sleep, anesthesia, and disorders of consciousness are shown in Fig. 1. These examples, which are clearly interesting in their own merit, are especially important for two reasons. First, because they share a common conceptual denominator in that they all tend to gauge the joint presence of functional integration and functional differentiation in the brain. Second, because they represent the tip of an iceberg of a much broader consilience, as we demonstrate below.

Overall, these works support the notion that the presence of consciousness is invariably associated with high brain complexity, which is found to be consistently decreased during deep sleep [Panel A, modified from (Hahn *et al.* 2021)], anesthesia [Panel B, modified from (Luppi *et al.* 2019)], and disorders of consciousness [Panel B, C, and D, modified from (Luppi *et al.* 2019), (King *et al.* 2013), and (Casarotto *et al.* 2016), respectively]. Notably, these studies employ different experimental approaches including both neurophysiological measures and functional brain imaging.

### A systematic literature analysis

In order to substantiate and characterize this emerging convergence, we performed a systematic search on the electronic database PubMed for original research studies published in journals and available online using the query: “consciousness” AND (complexity OR “entropy” OR “fractal” OR (“network” AND “graph”) OR “information transmission” OR “mutual information” OR “information flow” OR “information sharing” OR (“information” AND integration) OR “effective information” OR “effective connectivity” OR “criticality” OR “metastability”). These search criteria were defined in order to be specific for complexity-related metrics and to accommodate, at the same time, the variety of empirical research programmes (i.e. methods to estimate complexity and experimental approaches; Fig. 1).

After excluding from the results of this automatic search studies that are unrelated to the subject of measuring consciousness (e.g. clinical studies aimed at testing brain-based measures for anesthesia monitoring or for the neurophysiological assessment of partial seizures not involving loss of consciousness), studies applying complexity measures on other biological signals (e.g. heart rate variability), and studies exclusively based on modeling work and animal models, as well as works exclusively employing proprietary software whose algorithms are not publicly disclosed (e.g. bispectral index), we identified 182 original studies.

When systematically analyzed, the results of our search highlight several interesting elements supporting the idea of a strong, rapidly growing empirical convergence in the literature.

**Figure 2.**
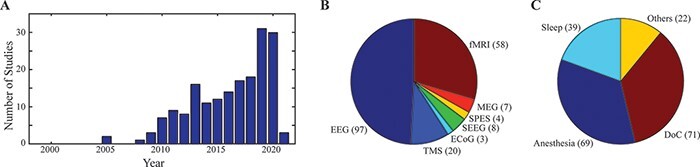
Literature on complexity-related measures

First, the identified studies cover a temporal span of more than a decade (the first paper identified dates 2005, Fig. 2A). Second, confirming the initial impression drawn from the examples provided in Fig. 1, this large body of empirical evidence encompasses studies employing a wide range of neurophysiological and neuroimaging tools (Fig. 2B), including scalp and intracranial EEG [either electrocorticography (ECoG) or stereo-EEG (SEEG)] alone or in combination with direct cortical perturbation [either transcranial magnetic stimulation (TMS) or single pulse electrical stimulation (SPES)] and magnetoencephalography (MEG), as well as functional magnetic resonance imaging (fMRI). Third, our search confirms that complexity-related measures have been successfully applied to index changes in the state of consciousness across a wide range of conditions (Tables 1–4), including sleep [both rapid eye movement (REM) and nonREM (NREM)], general anesthesia (employing various anesthetic agents with different mechanisms of action), meditation, and drug-induced altered states of consciousness, as well as neurological conditions, including epilepsy, coma, and disorders of consciousness (Fig. 2C). Remarkably, across this wide range of conditions and methodologies, the reliability of complexity-related measures was confirmed by all studies contrasting the presence versus the absence of consciousness. More specifically, consciousness was found invariably associated with high levels of brain complexity not only during wakefulness but also during REM sleep (Imperatori *et al.* 2020) and even in the extreme case of general anesthesia with ketamine (Sarasso *et al.* 2015). Conversely, brain complexity was found markedly reduced in all cases associated with the loss of consciousness whether during NREM sleep (Hahn *et al.* 2021), general anesthesia induced by the use of various compounds (Huang *et al.* 2016; Hashmi *et al.* 2017; Lee *et al.* 2020), or epileptic seizures (Mateos *et al.* 2018). Most importantly, in a number of studies involving the challenging conditions represented by the disorder of consciousness, the reliability of complexity-related measures in detecting consciousness was assessed explicitly by testing their specificity and sensitivity on large cohorts of patients. Notably, these experiments (Casali *et al.* 2013; Sitt *et al.* 2014; Casarotto *et al.* 2016; Engemann *et al.* 2018; Demertzi *et al.* 2019; Luppi *et al.* 2019) attained such a high accuracy in discriminating conscious from unconscious individuals that they were recently highlighted and formally recommended in a number of international practice guidelines (Giacino *et al.* 2018; Kondziella *et al.* 2020) and expert reviews (Bai *et al.* 2020; Comanducci *et al.* 2020) for the diagnosis of disorders of consciousness.

Panel A. The histogram represents the number of studies per year of publication (1998–2021) derived from our literature search. No study matched our search criteria before 2005. The total number of studies identified by our search is 182. Panel B. Number of studies for each of the different neurophysiological and neuroimaging tools employed. These included scalp and intracranial EEG (either EcoG or SEEG), alone or in combination with direct cortical perturbation (either TMS or SPES), MEG, as well as fMRI. Panel C. Number of studies for each of the different conditions in which complexity-related measures were applied to index changes in the state of consciousness. These included sleep, general anesthesia, disorders of consciousness, and other conditions where the state of consciousness is altered, such as meditation and drug-induced altered states of consciousness, as well as neurological conditions, including epilepsy (grouped together and labelled “Others” in the Figure).

**Table 1.** Papers assessing changes in the state of consciousness during general anesthesia

First author (year)	Journal abbreviation	DOI	Data type
Jordan (2008)	*Anesthesiology*	10.1097/ALN.0b013e31818d6c55	EEG
Lee (2009)	*Conscious Cogn*	10.1016/j.concog.2008.10.005	EEG
Ferrarelli (2010)	*Proc Natl Acad Sci USA*	10.1073/pnas.0913008107	TMS-EEG
Lee (2010)	*Anesthesiology*	10.1097/ALN.0b013e3181f229b5	EEG
Li (2010)	*J Neural Eng*	10.1088/1741-2560/7/4/046010	EEG
Kaskinoro (2011)	*Br J Anaesth*	10.1093/bja/aer196	EEG
Ku (2011)	*PloS One*	10.1371/journal.pone.0025155	EEG
Lee (2011)	*Anesthesiology*	10.1097/ALN.0b013e31821102c9	EEG
Schrouff (2011)	*NeuroImage*	10.1016/j.neuroimage.2011.04.020	fMRI
Barrett (2012)	*PloS One*	10.1371/journal.pone.0029072	EEG
Nicolaou (2012)	*PloS One*	10.1371/journal.pone.0033869	EEG
Schröter (2012)	*J Neurosci*	10.1523/JNEUROSCI.6046-11.2012	EEGfMRI
Casali (2013)	*Sci Transl Med*	10.1126/scitranslmed.3006294	TMS-EEG
Gili (2013)	*J Neurosci*	10.1523/JNEUROSCI.3480-12.2013	fMRI
Guldenmund (2013)	*Brain Connect*	10.1089/brain.2012.0117	fMRI
Jordan (2013)	*Anesthesiology*	10.1097/ALN.0b013e3182a7ca92	EEGfMRI
Kuhlmann (2013)	*PloS One*	10.1371/journal.pone.0056434	EEG
Lee (2013b)	*Anesthesiology*	10.1097/ALN.0b013e3182a8ec8c	EEG
Lee (2013c)	*Anesthesiology*	10.1097/ALN.0b013e31829103f5	EEG
Monti (2013)	*PloS Comput Biol*	10.1371/journal.pcbi.1003271	fMRI
Shin (2013)	*PloS One*	10.1371/journal.pone.0070899	EEG
Alonso (2014)	*Front Neural Circuits*	10.3389/fncir.2014.00020	SEEG
Liu (2014)	*PloS One*	10.1371/journal.pone.0092182	fMRI
Maksimow (2014)	*PloS One*	10.1371/journal.pone.0113616	EEG
Untergehrer (2014)	*PloS One*	10.1371/journal.pone.0087498	EEG
Liang (2015a)	*Hum Brain Mapp*	https://doi.org/10.1002/hbm.22914	fMRI
Liang (2015b)	*Clin Neurophysiol*	10.1016/j.clinph.2014.05.012	EEG
Moon (2015)	*PloS Comput Biol*	10.1371/journal.pcbi.1004225	EEG
Sarasso (2015)	*Curr Biol*	10.1016/j.cub.2015.10.014	TMS-EEG
Schartner (2015)	*PloS One*	10.1371/journal.pone.0133532	EEG
Casarotto (2016)	*Ann Neurol*	10.1002/ana.24779	TMS-EEG
Huang (2016)	*Neuroimage*	10.1016/j.neuroimage.2015.08.062	fMRI
Liang (2016)	*J Clin Monit Comput*	10.1007/s10877-015-9738-z	EEG
Blain-Moraes (2017)	*Front Hum Neurosci*	10.3389/fnhum.2017.00328	EEG
Crone (2017)	*Cereb Cortex*	10.1093/cercor/bhw112	fMRI
Hashmi (2017)	*Anesthesiology*	10.1097/ALN.0000000000001509	fMRI
Lee (2017a)	*Hum Brain Mapp*	10.1002/hbm.23708	EEG
Lee (2017b)	*Sci Rep*	10.1038/s41598-017-15082-5	EEG
Wang (2017)	*Neurosci Lett*	10.1016/j.neulet.2017.05.045	EEG
Eagleman (2018)	*Front Neurosci*	10.3389/fnins.2018.00645	EEG
Huang (2018)	*Br J Anaesth*	10.1016/j.bja.2018.04.031	SEEG
Kim (2018a)	*Front Hum Neurosci*	10.3389/fnhum.2018.00042	EEG
Kim (2018b)	*PloS Comput Biol*	10.1371/journal.pcbi.1006424	EEG
Lee (2018)	*Entropy*	10.3390/e20070518	fMRI
Li (2018)	*PloS One*	10.1371/journal.pone.0192358	fMRI
Afshani (2019)	*Cogn Neurodyn*	10.1007/s11571-019-09553w	EEG
Colombo (2019)	*Neuroimage*	10.1016/j.neuroimage.2019.01.024	EEGTMS-EEG
Comolatti (2019)	*Brain Stimul*	10.1016/j.brs.2019.05.013	TMS-EEGSPES-SEEG
Demertzi (2019)	*Sci Adv*	10.1126/sciadv.aat7603	fMRI
Eagleman (2019)	*PloS One*	10.1371/journal.pone.0223921	EEG
Golkowski (2019)	*Anesthesiology*	10.1097/ALN.0000000000002704	fMRI
Kim (2019)	*Entropy*	10.3390/e21100981	EEG
Lange (2019)	*Sci Rep*	10.1038/s41598-019-52949-1	EcoG
Lee (2019)	*NeuroImage*	10.1016/j.neuroimage.2018.12.011	EEG
Li (2019)	*NeuroImage*	10.1016/j.neuroimage.2019.03.076	EEG
Lioi (2019)	*Anaesthesia*	https://doi.org/10.1111/anae.14535	EEG
Liu (2019)	*Brain Imaging Behav*	10.1007/s11682-018-9886-0	fMRI
Luppi (2019)	*Nat Commun*	10.1038/s41467-019-12658-9	fMRI
Pappas (2019b)	*Anesthesiology*	10.1097/ALN.0000000000002977	EEG
Pappas (2019a)	*NeuroImage*	10.1016/j.neuroimage.2018.10.078	fMRI
Ruiz de Miras (2019)	*Comput Methods Programs Biomed*	10.1016/j.cmpb.2019.04.017	TMS-EEG
Wenzel (2019)	*Cell Syst*	10.1016/j.cels.2019.03.007	SEEG
Farnes (2020)	*PloS One*	10.1371/journal.pone.0242056	EEGTMS-EEG
Lee (2020)	*Sci Rep*	10.1038/s41598-020-59264-0	EEG
Liang (2020a)	*Anesthesiology*	10.1097/ALN.0000000000003015	EcoG
Pullon (2020)	*Anesthesiology*	10.1097/ALN.0000000000003398	EEG
Varley (2020c)	*Sci Rep*	10.1038/s41598-020-57695-3	fMRI
Wang (2020a)	*NeuroImage Clin*	10.1016/j.nicl.2020.102188	fMRI
Yan (2020)	*Clin EEG Neurosci*	10.1177/1550059420976303	EEG

**Table 2.** Papers assessing changes in the state of consciousness following severe brain injury

First author (year)	Journal abbreviation	DOI	Data type
Cauda (2009)	*J Neurol Neurosurg Psychiatry*	10.1136/jnnp.2007.142349	fMRI
Pollonini (2010)	*Brain Topogr*	10.1007/s10548-010-0139-9	EEG
Sarà (2010)	*Nonlinear Dynamics Psychol Life Sci*	PMID: 20021774	EEG
Gosseries (2011)	*Funct Neurol*	PMID: 21693085	EEG
Sarà (2011)	*Neurorehab Neural Rep*	10.1177/1545968310378508	EEG
Wu (2011)	*Clin Neurophysiol*	10.1016/j.clinph.2010.05.036	EEG
Zhou (2011)	*Conscious Cogn*	10.1016/j.concog.2010.08.003	fMRI
Achard (2012)	*Proc Natl Acad Sci U S A*	10.1073/pnas.1208933109	fMRI
Fingelkurts (2012)	*Open Neuroimag J*	10.2174/1874440001206010055	EEG
Rosanova (2012)	*Brain*	10.1093/brain/awr340	TMS-EEG
Casali (2013)	*Sci Transl Med*	10.1126/scitranslmed.3006294	TMS-EEG
Fingelkurts (2013)	*Clin EEG Neurosci*	10.1177/1550059412474929	EEG
King (2013)	*Curr Biol*	10.1016/j.cub.2013.07.075	EEG
Mäki-Marttunen (2013)	*Front Neuroinform*	10.3389/fninf.2013.00024	fMRI
Ragazzoni (2013)	*PloS One*	10.1371/journal.pone.0057069	TMS-EEG
Chennu (2014)	*PloS Comput Biol*	10.1371/journal.pcbi.1003887	EEG
Crone (2014)	*Neuroimage Clin*	10.1016/j.nicl.2013.12.005	fMRI
Liu (2014)	*PloS One*	10.1371/journal.pone.0092182	fMRI
Marinazzo (2014)	*Clin EEG Neurosci*	10.1177/1550059413510703	EEG
Sitt (2014)	*Brain*	10.1093/brain/awu141	EEG
Varotto (2014)	*Clin Neurophysiol*	10.1016/j.clinph.2013.06.016	EEG
Crone (2015)	*NeuroImage*	10.1016/j.neuroimage.2015.01.037	fMRI
Bai (2016)	*Front Neurosci*	10.3389/fnins.2016.00473	TMS-EEG
Casarotto (2016)	*Ann Neurol*	10.1002/ana.24779	TMS-EEG
Claassen (2016)	*Ann Neurol*	10.1002/ana.24752	EEG
Fingelkurts (2016)	*Open Neuroimag J*	10.2174/1874440001610010041	EEG
Huang (2016)	*Neuroimage*	10.1016/j.neuroimage.2015.08.062	fMRI
Kuceyeski (2016)	*Neuroimage Clin*	10.1016/j.nicl.2016.04.006	fMRI
Naro (2016)	*Brain Topogr*	10.1007/s10548-016-0489-z	EEG
Piarulli (2016)	*J Neurol*	10.1007/s00415-016-8196-y	EEG
Tagliazucchi (2016)	*J R Soc Interface*	10.1098/rsif.2015.1027	fMRI
Amico (2017)	*NeuroImage*	10.1016/j.neuroimage.2017.01.020	fMRI
Bodart (2017)	*Neuroimage Clin*	10.1016/j.nicl.2017.02.002	TMS-EEG
Chennu (2017)	*Brain*	10.1093/brain/awx163	EEG
Fingelkurts (2017)	*Clin EEG Neurosci*	10.1177/1550059417696180	EEG
Naro (2017)	*Neuroscience*	10.1016/j.neuroscience.2017.02.053	EEG
Wislowska (2017)	*Sci Rep*	10.1038/s41598-017-00323-4	EEG
Bodart (2018a)	*Brain Stimul*	10.1016/j.brs.2017.11.006	TMS-EEG
Cavaliere (2018)	*Front Neurol*	10.3389/fneur.2018.00861	fMRI
Dell’Italia (2018)	*Front Neurol*	10.3389/fneur.2018.00439	fMRI
Di Perri (2018)	*Hum Brain Mapp*	10.1002/hbm.23826	fMRI
Engemann (2018)	*Brain*	10.1093/brain/awy251	EEG
Rosanova (2018)	*Nat Commun*	10.1038/s41467-018-06871-1	TMS-EEG
Sinitsyn (2018)	*Hum Brain Mapp*	10.1002/hbm.24050	fMRI
Stefan (2018)	*Brain Topogr*	10.1007/s10548-018-0643-x	EEG
Wielek (2018)	*PloS One*	10.1371/journal.pone.0190458	EEG
Cacciola (2019)	*J Clin Med*	10.3390/jcm8030306	EEG
Comolatti (2019)	*Brain Stimul*	10.1016/j.brs.2019.05.013	TMS-EEGSPES-SEEG
Demertzi (2019)	*Sci Adv*	10.1126/sciadv.aat7603	fMRI
Lee (2019a)	*NeuroImage*	10.1016/j.neuroimage.2018.12.011	EEG
Luppi (2019)	*Nat Commun*	10.1038/s41467-019-12658-9	fMRI
Malagurski (2019)	*NeuroImage*	10.1016/j.neuroimage.2019.03.012	fMRI
Rizkallah (2019)	*NeuroImage Clin*	10.1016/j.nicl.2019.101841	EEG
Xia (2019)	*Neuroreport*	10.1097/WNR.0000000000001362	TMS-EEG
Abeyasinghe (2020)	*J Clin Med*	10.3390/jcm9051342	fMRI
Cai (2020b)	*J Neural Eng*	10.1088/1741-2552/ab79f5	EEG
Cai (2020a)	*J Neural Eng*	10.1088/1741-2552/ab8b2c	EEG
Carrière (2020)	*Brain Sci*	10.3390/brainsci10070469	EEG
Huang (2020)	*Neurocrit Care*	10.1007/s12028-020-01051w	EEG
Liang (2020b)	*IEEE Trans Neural Syst Rehabil Eng*	10.1109/TNSRE.2020.2964819	EEG
Lutkenhoff (2020)	*Brain Stimul*	10.1016/j.brs.2020.07.012	TMS-EEG
Martens (2020)	*Neuroimage Clin*	10.1016/j.nicl.2020.102426	EEG
Nadin (2020)	*Neurosci Conscious*	10.1093/nc/niaa017	EEG
Rudas (2020)	*Brain Connect*	10.1089/brain.2019.0716	fMRI
Sangare (2020)	*Brain Sci*	10.3390/brainsci10110845	EEG
Sinitsyn (2020)	*Brain Sci*	10.3390/brainsci10120917	TMS-EEG
Varley (2020b)	*PloS One*	10.1371/journal.pone.0223812	fMRI
Wang (2020b)	*Int J Neurosci*	10.1080/00207454.2019.1702543	EEG
Wu (2020)	*Entropy*	10.3390/e22121411	EcoG
Zhang (2020)	*Front Hum Neurosci*	10.3389/fnhum.2020.560586	EEG
Naro (2021)	*Int J Neural Syst*	10.1142/S0129065720500525	EEG

**Table 3.** Papers assessing changes in the state of consciousness during sleep

First author (year)	Journal abbreviation	DOI	Data type
Burioka (2005)	*Clin EEG Neurosci*	10.1177/155005940503600106	EEG
Massimini (2005)	*Science*	10.1126/science.1117256	TMS-EEG
Massimini (2010)	*Cogn Neurosci*	10.1080/17588921003731578	TMS-EEG
Spoormaker (2010)	*J Neurosci*	10.1523/JNEUROSCI.2015-10.2010	fMRI
Boly (2012)	*Proc Natl Acad Sci U S A*	10.1073/pnas.1111133109	fMRI
Chu (2012)	*J Neurosci*	10.1523/JNEUROSCI.5669-11.2012	EEG
Casali (2013)	*Sci Transl Med*	10.1126/scitranslmed.3006294	TMS-EEG
Lee (2013a)	*Front Neuroinform*	10.3389/fninf.2013.00033	EEG
Tagliazucchi (2013)	*Proc Natl Acad Sci U S A*	10.1073/pnas.1312848110	fMRI
Zorick (2013)	*PloS One*	10.1371/journal.pone.0068360	EEG
Uehara (2014)	*Cereb Cortex*	10.1093/cercor/bht004	EEG fMRI
Allegrini (2015)	*Phys Rev E*	10.1103/PhysRevE.92.032808	EEG
Pigorini (2015)	*NeuroImage*	10.1016/j.neuroimage.2015.02.056	SPES-SEEG
Usami (2015)	*Hum Brain Mapp*	10.1002/hbm.22948	SPES-EcoG
Andrillon (2016)	*J Neurosci*	10.1523/JNEUROSCI.0902-16.2016	EEG
Casarotto (2016)	*Ann Neurol*	10.1002/ana.24779	TMS-EEG
Guevara Erra (2016)	*Phys Rev E*	10.1103/PhysRevE.94.052402	EEGSEEGMEG
Tagliazucchi (2016)	*Brain Struct Funct*	10.1007/s00429-015-1162-0	fMRI
Lioi (2017)	*Physiol Meas*	10.1088/1361-6579/aa81b5	EEG
Schartner (2017b)	*Neurosci Conscious*	10.1093/nc/niw022	SEEG
Wislowska (2017)	*Sci Rep*	10.1038/s41598-017-00323-4	EEG
Isler (2018)	*PloS One*	10.1371/journal.pone.0206237	EEG
Li (2018)	*PloS One*	10.1371/journal.pone.0192358	fMRI
Mateos (2018)	*Cogn Neurodyn*	10.1007/s11571-017-9459-8	EEGSEEGMEG
Rosanova (2018)	*Nat Commun*	10.1038/s41467-018-06871-1	TMS-EEG
Wielek (2018)	*PloS One*	10.1371/journal.pone.0190458	EEG
Bocaccio (2019)	*J R Soc Interface*	10.1098/rsif.2019.0262	fMRI
Comolatti (2019)	*Brain Stimul*	10.1016/j.brs.2019.05.013	TMS-EEGSPES-SEEG
Imperatori (2019)	*Sci Rep*	10.1038/s41598-019-45289-7	EEG
Kung (2019)	*Hum Brain Mapp*	10.1002/hbm.24590	EEG fMRI
Lee (2019b)	*Sci Rep*	10.1038/s41598-019-41274-2	TMS-EEG
Miskovic (2019)	*Hum Brain Mapp*	10.1002/hbm.24393	EEG
Ruiz de Miras (2019)	*Comput Methods Programs Biomed*	10.1016/j.cmpb.2019.04.017	TMS-EEG
Usami (2019)	*Sleep*	10.1093/sleep/zsz050	SPES-EcoG
Frohlich (2020)	*Neurosci Conscious*	10.1093/nc/niaa005	EEG
Hou (2020)	*Sleep*	10.1093/sleep/zsaa226	EEG
Imperatori (2020)	*Sleep*	10.1093/sleep/zsaa247	EEG
Wang (2020)	*Neuroimage Clin*	10.1016/j.nicl.2020.102188	fMRI
Hahn (2021)	*NeuroImage*	10.1016/j.neuroimage.2020.117470	fMRI

**Table 4.** Papers assessing changes in the state of consciousness during epileptic seizures and other conditions (e.g. psychedelics, meditation)

First author (year)	Journal abbreviation	DOI	Data type
Epilepsy			
Vaudano (2009)	*PloS One*	10.1371/journal.pone.0006475	EEG fMRI
Song (2011)	*PloS One*	10.1371/journal.pone.0017294	fMRI
Li (2015)	*J Neurol Sci*	10.1016/j.jns.2015.04.054	fMRI
Guevara Erra (2016)	*Phys Rev E*	10.1103/PhysRevE.94.052402	EEGSEEGMEG
Mateos (2017)	*Phys Rev E*	10.1103/PhysRevE.96.062410	EEGSEEG MEG
Mateos (2018)	*Cogn Neurodyn*	10.1007/s11571-017-9459-8	EEG SEEG MEG
Dheer (2020)	*Heliyon*	10.1016/j.heliyon.2020.e05769	SEEG
Psychedelics			
Tagliazucchi (2014)	*Hum Brain Mapp*	10.1002/hbm.22562	fMRI
Lebedev (2015)	*Hum Brain Mapp*	10.1002/hbm.22833	fMRI
Palhano-Fontes (2015)	*PloS One*	10.1371/journal.pone.0118143	fMRI
Schartner (2017a)	*Sci Rep*	10.1038/srep46421	MEG
Viol (2017)	*Sci Rep*	10.1038/s41598-017-06854-0	fMRI
Preller (2019)	*Proc Natl Acad Sci U S A*	10.1073/pnas.1815129116	fMRI
Viol (2019)	*Entropy*	10.3390/e21020128	fMRI
Barnett (2020)	*NeuroImage*	10.1016/j.neuroimage.2019.116462	MEG
Varley (2020a)	*NeuroImage*	10.1016/j.neuroimage.2020.117049	fMRI
Luppi (2021)	*NeuroImage*	10.1016/j.neuroimage.2020.117653	fMRI
Meditation			
Panda (2016)	*Front Hum Neurosci*	10.3389/fnhum.2016.00372	EEG fMRI
Escrichs (2019)	*Front Syst Neurosci*	10.3389/fnsys.2019.00027	fMRI
Dürschmid (2020)	*PLoS One*	10.1371/journal.pone.0233589	MEG
Fetal and neonatal			
Moser (2019)	*Front Syst Neurosci*	10.3389/fnsys.2019.00023	MEG
Mechanical stimulation of the olfactory system			
Piarulli (2018)	*Sci Rep*	10.1038/s41598-018-24924-9	EEG

Last but not least, it is worth noting that, overall, this large body of empirical evidence was accumulated by clinicians and researchers from several independent research groups encompassing a wide spectrum of theoretical views.

## Consciousness and complexity: rewinding 20 years

Prompted by the consistent results highlighted in the previous section, we here revisit the original rationale for explicitly linking consciousness to complexity. In doing so, we identify a set of principles and predictions that can help interpret and conceptually organize the broad spectrum of current methods and measures.

### Early theoretical principles

The first clear landmark linking consciousness to complexity dates back to more than 20 years ago, when a paper explicitly titled “Consciousness and complexity” was published in *Science* (Giulio Tononi and Edelman 1998). Here, Tononi and Edelman defined the kind of complexity specifically relevant for consciousness as the coexistence of a high degree of functional differentiation and functional integration within a system, conceiving a testable proposal regarding the neural substrate of conscious experience called the dynamic core hypothesis (DCH).

Using phenomenology as a springboard, the DCH combined the two ingredients of integration and differentiation in a novel framework about the relationships between consciousness and the brain. As described at the outset of the original paper, a simple exercise of introspection indicates that each experience is both integrated (each conscious scene is unified) and highly differentiated (each experience is specific, differing from a huge number of different conscious states at any given time). Thus, the neural process underlying conscious experience must be functionally integrated and, at the same time, highly differentiated. Starting from this premise, the DCH postulated which basic anatomical and neurophysiological properties may be specifically relevant for consciousness.

The first necessary property is a pattern of structural connectivity characterized by the coexistence of functional segregation and functional integration. Besides the appropriate arrangement of structural connectivity, the DCH postulated that another necessary property is the presence of effective and rapid reentrant interactions, granting the formation of a tightly integrated cluster of neurons. Perhaps the most interesting (and defining) claim of Tononi and Edelman is that none of the above is a sufficient property for conscious experience and that only if the integrated cluster is capable of a large repertoire of different states, consciousness is possible.

In a nutshell, the backbone of the DCH consisted in the following two principles

- A group of neurons can contribute directly to conscious experience only if it is part of a distributed functional cluster [i.e. a subset of a neural system with dynamics that display high statistical dependence internally and relatively lower dependence with elements outside the subset (Tononi *et al.* 1998)] that achieves high integration in hundreds of milliseconds.
- To sustain conscious experience, it is essential that this functional cluster is capable of a large repertoire of different activity patterns or neural states, as indicated by high values of complexity.

### Early theoretical measures

Even more importantly in the present context, Tononi and Edelman proposed a metric, called neural complexity (C_N_), to quantify the above-mentioned physiologically observable variables (balanced structural connectivity, integration through fast reentrant interactions, and the differentiation of neuronal activity) in thalamocortical networks. The fundamental insight offered by the DCH and by the related measure C_N_ is that functional integration and functional differentiation need to be measured jointly. A basic concept, with key practical implications, is that measuring the spatial extent of large-scale synchronous dynamics would not suffice *per se*. Indeed, one would not take into account whether these integrated dynamics are differentiated or stereotypical (such as the ones recorded during unconscious seizures or NREM sleep). On the other hand, according to the DCH, simply measuring the algorithmic complexity or the entropy of ongoing time series would not do, which also has practical implications. In this case, in fact, one would not know whether these differentiated patterns are generated by one system of interacting elements or by a collection of independent elements.

Following the intuitions of the DCH and further specifying the concepts of integration and differentiation, Tononi and colleagues later introduced a novel measure called Փ (Tononi 2001; Tononi and Sporns 2003). A critical difference is that this measure is based on *effective* rather than *mutual* information (the core measure of C_N_), thus reflecting *causal interactions* rather than *statistical dependencies*. A first implication is that, through extensive perturbations of all the subsets of the system, Փ captures not only the states the system cycles through within the limited time of observation but all the potential states the system is capable of (i.e. its full repertoire of states). Perhaps more importantly, this perturbational approach bears practical consequences for the development of empirical metrics. Indeed, the strategy of measuring causal interactions instead of temporal correlations takes the problem of measuring integration (i.e. the unity of a system) very seriously, as it avoids confounds due to spurious sources of integration such as common drivers and correlated inputs.

Given the obvious computational burden of both C_N_ and Փ, parallel attempts were made in order to develop more applicable measures. In this context, causal density (cd) was proposed by Seth and colleagues (Seth 2005) as a viable method to measure the coexistence of differentiation and integration, which they called the “relevant complexity” (Seth *et al.* 2006). By leveraging the econometric concept of Granger causality, a measure of causal influence based on time-series inference, cd measures the overall causal interactivity sustained by a system. Specifically, cd captures the dynamical heterogeneity among network elements (differentiation) as well as their global dynamical integration. Given that the number of parameters to be estimated is lower with respect to both C_N_ and Փ, cd is, in principle, more tractable. Potential drawbacks of cd are that it estimates causal interaction only from an observational perspective and that its reliability can be significantly affected by time-series nonstationarity.

In the following years, these measures were further refined based on both theoretical and practical considerations (Barrett and Seth 2011; Toker and Sommer 2019). Yet, the original formulation of C_N_, Փ, and cd represents the first attempt at measuring a new kind of complexity and a valid reference for our analysis of the empirical metrics that were subsequently applied.

### Early theoretical predictions

Besides delineating a novel trajectory from phenomenology to a new class of measures, as described above, the DCH framework also laid specific predictions on the relationship between consciousness and brain complexity. Specifically

- The structural architecture (high density of connections, strong local connectivity, patchiness in the connectivity among neuronal groups, and large numbers of short reentrant circuits) of certain brain regions will be much more effective in generating high complexity than that of other regions. It follows that at least some regions of the thalamocortical system are endowed with the specific anatomical requirements supporting conscious experience, while others, such as the cerebellum or the basal ganglia, are not.
- Altering these anatomical requirements, as in the case of brain lesions involving thalamocortical networks leading to disorders of consciousness, will result in a decrease in complexity.
- Anatomical requirements being equal, changes in functional neuronal properties that affect reentry, integration, and differentiation will result in the loss of complexity and, in turn, of consciousness in conditions such as NREM sleep, general anesthesia, and generalized seizures.
- High complexity and conscious experience can be supported by intrinsic brain interactions even in the absence of sensory inputs and motor outputs. As such, complexity will be high during REM sleep (a state of sleep in which subjects almost always dream) and in other disconnected states.

The empirical test of this set of predictions was initially hampered by limitations inherent to human recording/imaging techniques and by a lower computational power at the time. However, as soon as the appropriate equipment and analysis tools became available, the accumulation of relevant empirical data accelerated and complexity measures were endorsed by an increasing number of scholars (Alkire *et al.* 2008; Sitt *et al.* 2013; Ruffini 2017; Aru *et al.* 2020) in conjunction with an obvious increase of original works in the last decade (Fig. 2). Due to a considerable gap between the time of the original predictions and the moment a critical amount of relevant data became available (almost 20 years, as shown in Fig. 2A), it is understandable that the two events are not always connected explicitly. This gap, however, represents an interesting opportunity, in at least two respects. On the one hand, recent experiments can be almost considered as an independent set of validation tests for early predictions. On the other, the original core concept of integration and differentiation can be employed as an objective principle to analyze current empirical approaches to measuring brain complexity.

## Consciousness and complexity: a provisional taxonomy

In the previous section, we reappraised the original rationale for linking consciousness to complexity. According to this proposal, the complexity that is relevant for consciousness seems to be a matter of balance, a balance between diversity and unity, or, more technically, the amount/repertoire of differentiated states (diversity) that can be generated by a set of interacting elements (unity). At least in principle, in order to quantify complexity, one should know the exact structure of the interactions within a system. This becomes obviously impossible when it comes to real brains, and empirical approximations are warranted. Our survey of the literature reveals that current empirical complexity-related measures have addressed this problem according to different strategies.

Recognizing that other schemes of classifications might be possible, we here provide a provisional taxonomy of this large body of literature along two main dimensions that we think are conceptually and practically useful.

The first dimension relates to the problem of estimating the repertoire of differentiated states available to a system. Here, we identify two main approaches adopted by different studies, which mainly differ in the way they treat brain activity time series. We then report their relevant empirical intuitions, define their rationale, and highlight their operational aspects. Finally, we identify a number of works proposing interesting mixed approaches.

The second dimension addresses the problem of constraining the repertoire of states only to those generated by interactions within the system, i.e. the problem of properly assessing the integration or the unity of the system by evaluating its internal interactions from a causal perspective.

### Estimating the repertoire of states. A first strategy: topological differentiation

Let us start with the first dimension of the problem, i.e. the estimation of the repertoire of states. A first strategy adopted for estimating this repertoire makes use of time series in order to extract the topological properties of the underlying network, the complexity of which is then captured by measures of segregation and integration. We identify the core ingredients (see below) of this strategy in 102 publications included in our literature search (Supplementary Table S1).

The underlying rationale is that the ability of a system to generate a large repertoire of states should correspond to topological features characterized by functionally segregated and densely connected modules that are at the same time integrated through longer-range, sparse connections.

**Figure 3.**
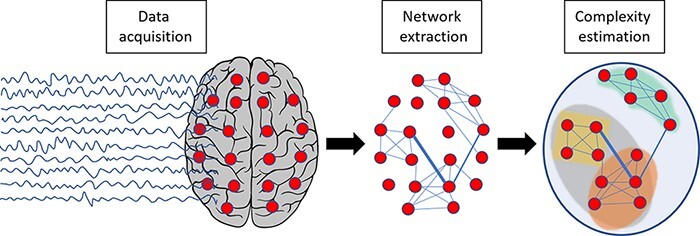
Schematic representation of the first strategy for estimating the repertoire of brain states (topological differentiation)

Operationally, this first strategy entails a two-step procedure: (i) the extraction of a network of interacting elements from empirical data and (ii) the estimation of the balance between segregation and integration within this network (Fig. 3).

A network of interacting elements is extracted from empirical data (M/EEG or fMRI time series) using different methods (e.g. temporal correlations, coherence, phase synchronization or measures of information flow). Complexity is then estimated on the extracted network by applying different metrics of segregation and integration, such as measures of network modularity, density, node centrality, clustering coefficient, local and global efficiency, and small-worldness, or by applying functional connectivity, entropic, algorithmic or fractal indices.

1. Network extraction: network elements or nodes are defined at different temporal resolutions (from milliseconds to seconds) and at different spatial scales (from groups of neurons to macroareas and functional subnetworks) depending on the adopted investigational techniques (from invasive to noninvasive M/EEG techniques and fMRI). Network edges (i.e. the relationships between interacting nodes) are identified by employing different metrics, such as temporal correlations, coherence, phase synchronization, and measures of information flow (Barnett *et al.* 2020; Nadin *et al.* 2020).
2. Complexity estimation: the majority of studies estimate the balance between segregation and integration by employing metrics of graph theory, such as measures of network modularity, density, node centrality, clustering coefficient, local and global efficiency, and small-worldness (Chennu *et al.* 2017; Luppi *et al.* 2021). Others use functional connectivity techniques that allow for the quantification of connections both within and between different functional subnetworks (Schrouff *et al.* 2011). Finally, the spatial complexity of connections between elements, as measured by entropic, algorithmic, and fractal indices, can also be employed to gauge the balance of local segregation and global integration within the reconstructed network (Viol *et al.* 2019).

Notably, some studies only perform the first step and characterize the connectivity between distributed neuronal groups without explicitly accounting for the balance between the segregation and integration of the underlying network (Liang *et al.* 2016; Huang *et al.* 2018). Although valuable from a descriptive point of view, these approaches are less explicitly related to brain complexity.

### Estimating the repertoire of states. A second strategy: temporal differentiation

An alternative strategy (adopted by 86 publications derived from our literature search; Supplementary Table S1) is to directly estimate complexity based on the amount of information (or differentiation) encoded in the temporal dynamics of brain activations.

The underlying rationale is that the size of the repertoire of available states is a function of the number of nonredundant patterns generated during the temporal evolution of the system.

**Figure 4.**
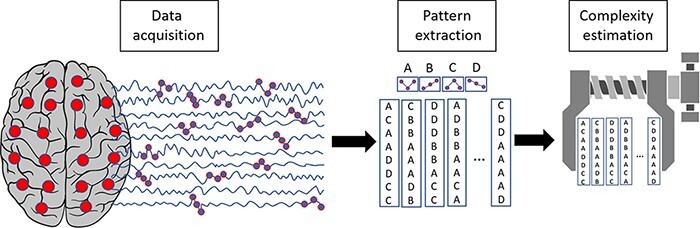
Schematic representation of the second strategy for estimating the repertoire of brain states (temporal differentiation)

Operationally, this second strategy entails a two-step procedure: (i) the extraction of temporal patterns of activity that result from the interactions among neuronal groups and (ii) the estimation of the complexity or information content of the resulting time series (Fig. 4).

Patterns or states of brain activation are extracted directly from brain activity time series or by applying a variety of methods such as signal binarization or symbolization, signal transformations (e.g. principal components and surface Laplacian), embedding or cluster analysis. Complexity is then estimated on the extracted temporal patterns by means of entropy measures, fractal measures, indexes of algorithmic complexity, number and distribution of microstates, methods based on recurrence quantification analysis, and empirical approximations of integrated information (Փ) or by applying measures of information sharing, such as mutual information or transfer entropy.

Pattern extraction: different studies define patterns or states of brain activation in a variety of ways and at multiple spatiotemporal scales depending on the employed techniques. Patterns can be identified as absolute signal fluctuations (Tagliazucchi *et al.* 2013) or by applying methods such as signal binarization or symbolization, signal transformations (e.g. principal components and surface Laplacian), embedding, or cluster analysis (Demertzi *et al.* 2019; Wenzel *et al.* 2019).

Complexity estimation: once activation patterns have been identified, a variety of metrics can be used to quantify temporal complexity, such as entropies, fractal measures, indexes of algorithmic complexity, number and distribution of microstates, methods based on recurrence quantification analysis, and empirical approximations of integrated information (Փ). Examples of such applications can be found in Andrillon *et al.* (2016), Mateos *et al.* (2018), and Liu *et al.* (2019). Measures of information sharing, such as mutual information or transfer entropy (which are more typically used to construct the edges between nodes in a graph, as in the first strategy), can also be employed as indexes of the amount of information exchanged by interacting brain regions (King *et al.* 2013).

It is worth noting that, also in this case, a small subset of studies (see, e.g. Massimini *et al.* 2005; Usami *et al.* 2015) only performed the first step explicitly, thus quantifying the differentiation only in a qualitative manner (i.e. without formally applying quantitative measures to estimate complexity).

### Estimating the repertoire of states. A mixed strategy and its relationships to metastability and criticality

**Figure 5.**
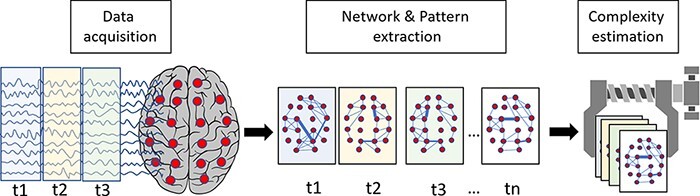
Schematic representation of the mixed strategy for estimating the repertoire of brain states

A small number of studies (23 in our literature search) adopted a combination of the above-mentioned strategies in that they make use of time series in order to construct the topological properties of the underlying network (as in the first strategy) and then quantify their differentiation over time (as in the second strategy). Operationally, this approach generally works by employing relatively short temporal sliding windows in order to construct a time series of quasi-stable network configurations, which is then used to estimate the repertoire of states available to the system (Fig. 5). Examples of such mixed strategy can be found in Golkowski *et al.* (2019) and Cai *et al.* (2020a).

This strategy adopts a combination of the other two in that it extracts topological properties from empirical data (as in the first strategy) and then quantifies their differentiation over time (as in the second strategy).

It is interesting to note that the high variability of such quasi-stable configurations or quasi-stationary states is also considered a mark of metastable regimes, where the system is out of equilibrium and is driven to visit different states by intrinsic or extrinsic events (Tognoli and Kelso 2014; Deco *et al.* 2017; Cavanna *et al.* 2018). Some of the studies in our literature search make explicit use of such concepts and estimate complexity by the level of integration of different neuronal assemblies during events such as rapid transition periods (Fingelkurts *et al.* 2013) or intrinsic ignitions across brain areas (Deco *et al.* 2017; Escrichs *et al.* 2019; Hahn *et al.* 2021). Metastability is, in turn, related to the phenomena of criticality and phase transition in dynamical systems: the variability of states or network configurations is generally maximized at critical values of specific parameters controlling the dynamics of the system. In subcritical regimes, interactions among the elements become dominant and the system tends toward complete order. At the other extreme, in supercritical regimes, external sources and local fluctuations dominate the dynamics and the system fragments into a multitude of independent clusters. The balance between order (integration) and disorder (segregation) can thus also be estimated by markers of criticality, such as measures of scale-free behaviors (Allegrini *et al.* 2015; Bocaccio *et al.* 2019; Colombo *et al.* 2019; Dürschmid *et al.* 2020), hysteresis (Kim *et al.* 2018a), and dynamical instability (Alonso *et al.* 2014).

### Estimating integration: observation vs perturbation

Irrespective of the strategy adopted to estimate the repertoire of states, one key aspect relates to the methods adopted to assess integration, i.e. to constrain such repertoire to those states that are generated by genuine interactions among neuronal groups.

This is important as it tackles the problem of distinguishing between a unitary system made of tightly interacting elements and an aggregate of largely independent generators of activity. Adopting a causal perspective is a viable way to approach this issue and best approximate the structure of the interactions within a system, which, as discussed above, represents a key prerequisite to quantify the relevant complexity. In practical terms, measuring causal relationships as opposed to statistical dependencies contrasts the influence of spurious sources of integration, such as common drivers and correlated inputs, and minimizes the influence of noise, which can artificially affect complexity estimations (e.g. in the presence of random patterns).

The ideal way to do so is to perturb the system in a controlled manner in order to reliably establish causes and effects (Paus 2005; Pearl 2009; Pearl and Mackenzie 2018). However, the vast majority of studies (159 in our literature search; see Supplementary Table S1) adopted an observational strategy, often assisted by specific methods to mitigate the risk of confusing correlation with causation (Friston 2011). Some of these are aimed at diminishing activities and patterns originating from sources other than interacting neuronal elements. Examples of such applications are methods of signal decomposition, surface Laplacian, and source reconstruction to reduce spurious, non-neural correlations (Schartner *et al.* 2017b); symbolic and multiscale strategies to reduce the impact of noise and random fluctuations (Lee *et al.* 2018); and the use of measures such as weighted symbolic mutual information, which is based on disregarding co-occurrences of identical or opposite-sign patterns in order to reduce the effects of common drivers (King *et al.* 2013). Statistical measures of directed connectivity, such as transfer entropy and Granger causality, both in time and frequency domain, are frequently used as probabilistic accounts of actual causation (Barrett *et al.* 2012). Correlating temporal complexity with structural connectivity or with the activity of known integrated functional networks is another way to substantiate the causal origin of the observed information (Tagliazucchi *et al.* 2013; Tagliazucchi 2016). Finally, dynamic causal modeling was also applied to the problem of consciousness, although this approach is more commonly used to characterize the type and strength of interactions across a small number of brain areas rather than estimating complexity (Vaudano *et al.* 2009; Preller *et al.* 2019).

**Figure 6.**
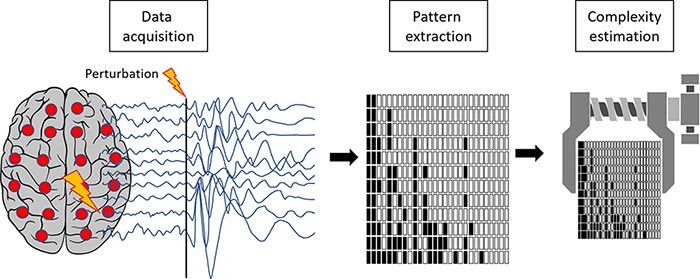
A perturbational approach to brain complexity

On the other hand, only a small number of studies (23 in our literature search, see Supplementary Table S1) assessed the causal structure of a system by analyzing its responses to direct cortical perturbations, which enable extracting only the patterns of activity that are generated through effective interactions. Operationally, these studies employed a “perturb and measure” approach based on the use of noninvasive techniques, such as navigated TMS and high-density electroencephalography (hd-EEG), as well as invasive intracranial electrical stimulations and recordings (Massimini *et al.* 2005; Ragazzoni *et al.* 2013; Pigorini *et al.* 2015; Usami *et al.* 2019; Sinitsyn *et al.* 2020). In particular, a subset of these studies, explicitly inspired by theoretical principles (Tononi 2004), combined this perturbational approach with strategies such as nonparametric statistical analysis and algorithmic complexity at the sources level (Casali *et al.* 2013) or principal component decomposition and recurrence quantification analysis at the sensors level (Comolatti *et al.* 2019), yielding indices of “perturbational complexity” (or PCI—Perturbational Complexity index; Fig. 6).

The causal structure of a system is assessed by applying local direct cortical perturbations, and complexity is estimated by retaining only the patterns of activity that are generated through effective interactions engendered by the perturbation.

Far from being definitive, this taxonomic attempt illustrates the wide range of approaches that have been adopted to estimating brain complexity, highlights their relationship, and identifies some of their caveats. In this latter respect, our analysis identified instances in which measures of differentiation were applied to signals obtained from single brain regions or recording channels. These metrics, albeit practically useful, necessarily rely on the strong assumption that the degree of differentiation they quantify reflects properties of the system as a whole (i.e. they take integration for granted). Clearly, in these cases, the estimation of the relevant complexity can be confounded by patterns produced by independent neural generators or noise. On the other hand of the spectrum, we found measures that were solely based on global spatiotemporal correlations. These measures, which do not explicitly account for differentiation, can provide high values for patterns that are highly correlated but stereotypical and not complex. In between these two extremes, the vast majority of the identified works (133 out of 182) employed methods to estimate both integration and differentiation. This recent trend provides strong evidence for a shared commitment to develop practical indices of consciousness that gauge the balance between diversity and unity in the brain in noticeable agreement with early theoretical proposals.

## Consciousness and complexity: the next 20 years

In the previous sections, we have highlighted an interesting trajectory traversing the science of consciousness over the last two decades. The emerging consilience is that measures of complexity, all designed to capture the joint presence of integration and differentiation in the brain, represent reliable indices of the state of consciousness. Starting from this common empirical background, we now consider open issues of both practical and theoretical relevance that should be addressed in the years to come in order to further advance this promising front.

### Complexity and the capacity for consciousness

Assessing sensitivity and specificity across different conditions is the ultimate practical guide to judge how a given marker approximates neuronal processes that are relevant to the presence or absence of consciousness. While some studies assessed the performance of complexity-related metrics only at the group level and others provided a precise quantification of their accuracy in a clinical setting, they all concurred on the same conclusion: complexity is higher in conditions in which consciousness is present and lower in conditions where this is lost. Such a high degree of consistency across different conditions, including challenging cases, such as disconnected consciousness in dreaming and ketamine anesthesia, hallucinatory states, and patients with severe brain injuries, is remarkable when compared to the discrepancies characterizing other classes of measures.

For example, when directly compared, complexity measures largely outperform ERPs, such as the P3b, in the detection of minimally conscious patients, the latter being characterized by lower sensitivity (Sitt *et al.* 2014). Also, complexity remains high in conscious subjects during REM sleep or ketamine hallucinations (Casarotto *et al.* 2016; Farnes *et al.* 2020), whereas ERPs to global deviant stimuli typically disappear (Strauss *et al.* 2015; Bravermanová *et al.* 2018). Likewise, complexity measures consistently show high values and dissociate from EEG alpha power, which is known to decrease in conditions of disconnected consciousness, such as during dreaming, hallucinations, and in the locked-in syndrome (Esposito *et al.* 2004; Babiloni *et al.* 2010; Timmermann *et al.* 2019). Measuring brain complexity also overcomes some of the limitations of indices quantifying scalp EEG delta power; indeed, wakefulness-like complexity values can be detected even when high-amplitude slow waves dominate the spontaneous scalp EEG in some minimally conscious patients (Casarotto *et al.* 2016) as well as in conscious children with Angelman syndrome (Frohlich *et al.* 2020) and in healthy conscious subjects administered with the GABA reuptake inhibitor tiagabine (Darmani *et al.* 2021). On the other hand, gamma band synchrony [an early proposed neural correlate of consciousness (Crick and Koch 1990)] and brain metabolism can persist or even increase during epileptic seizures (Pockett and Holmes 2009; Bai *et al.* 2010), whereas complexity is found invariably reduced (see, e.g. Song *et al.* 2011; Mateos *et al.* 2018), consistent with the loss of consciousness characterizing this condition. Overall, it seems that complexity is more reliable than other metrics, arguably offering not only better diagnostic accuracy but also a potential guide to identify, among the many facets of brain activity, core properties that are more relevant to consciousness.

Clearly, discrepancies will be found also in the case of complexity-related measures. As we will discuss later, this is to be expected if only one considers the number of different methods and spatiotemporal scales at which complexity is estimated. The taxonomy described in the previous section is meant to provide a framework to help interpret potentially emerging conflicting results and to select the most promising set of measurement strategies. Toward this aim, it will be critical for future studies to precisely identify how they define and quantify complexity and to explicitly report each measure’s performance in terms of sensitivity and specificity whenever a ground-truth about the state of consciousness of individual subjects is available (Demertzi *et al.* 2017).

Practically, to the extent that they have been extensively validated in benchmark conditions, indices of brain complexity can be effectively employed analogously to other scalar measures that are normally employed in medicine. Just like measuring the ejection fraction provides a rough but useful index of the heart’s capacity to sustain hemodynamic functions, measuring complexity may provide a rough but useful index to infer the brain’s ability to sustain consciousness. For example, detecting wakefulness-like complexity levels in the brain of a patient who is behaviorally unresponsive and inaccessible through ERPs indicates that this patient is disconnected on the output and/or input side rather than unconscious (Casarotto *et al.* 2016; Rohaut *et al.* 2017; Bayne *et al.* 2020a). Whether more subtle, monotonically varying changes in brain complexity are meaningful with respect to graded changes in conscious states is an open question (Cecconi *et al.* 2020; Bayne *et al.* 2020b). Answering this question would require not only performing measurements at the optimal spatiotemporal scale (see more about this below) but also a shared notion of levels of consciousness (Bayne *et al.* 2016; Mckilliam 2020). In this concern, early proposals have provisionally defined empirical measures of the coexistence of integration and differentiation as indices of the “capacity for” rather than of the “level of” consciousness (Massimini *et al.* 2009). As already noted above, this notion entails the general capacity for any kind of experience that is not necessarily associated with the cognitive operations typical of wakefulness. Indeed, high brain complexity can be detected in conscious conditions where the functional requirements of wakefulness are relaxed or absent, such as during dreaming and ketamine anesthesia (Sarasso *et al.* 2015; Casarotto *et al.* 2016).

### Mechanisms of loss and recovery of complexity

As loss and recovery of consciousness can be reliably tracked by shifts in brain complexity in humans, it is crucial to explore the mechanisms of these changes at the neuronal level. For example, one may ask why, as multiple studies have demonstrated, complexity collapses when consciousness fades during sleep and anesthesia, structural connections remaining equal. Interestingly, recent studies have drawn connections between specific changes in postsynaptic properties of single neurons and the brain capacity for engaging in complex patterns of interactions. A first candidate mechanism, called neuronal bistability, is the tendency of cortical neurons to plunge into a silent, hyperpolarized state (OFF-period) upon receiving an input (Steriade *et al.* 1993). This tendency is due to adaptation mechanisms, which become prominent when activity-dependent potassium currents are stronger (Sanchez-Vives and McCormick 2000) or when the excitation/inhibition balance is shifted toward the latter (Funk *et al.* 2017). A wealth of animal studies has shown the occurrence of neuronal OFF-periods during sleep and anesthesia, where they are associated with the appearance of EEG slow waves (Steriade *et al.* 1993; Sanchez-Vives *et al.* 2017). Crucially, converging noninvasive and intracranial works in humans, rodents, and cortical slices (D’Andola *et al.* 2018; Comolatti *et al.* 2019; Dasilva *et al.* 2021) have recently demonstrated that neuronal OFF-periods determine a dramatic collapse of brain complexity (loss of both integration and differentiation), as measured by PCI, during NREM sleep and anesthesia. Perhaps even more relevant in the present context, a direct link between bistability and loss of complexity has also been demonstrated in brain-injured humans (Rosanova *et al.* 2018); not only the buildup of causal interactions and complexity is blocked by OFF-periods in unconscious patients but also both global brain complexity and consciousness recover when OFF-periods disappear.

In light of the above, it is not surprising that conditions characterized by low brain complexity tend to be associated with the presence of slow waves, as both are linked to neuronal bistability. However, interesting dissociations are also possible. For example, EEG macroscale recordings can be at once characterized by a predominant delta spectral power and preserved complexity (Frohlich *et al.* 2020; Darmani *et al.* 2021). This apparent paradox might be explained by a mixed pattern at the mesoscale level where focal but powerful cortical sources of slow waves emerge in a brain that is otherwise in a state of wake-like complexity (Frohlich *et al.* 2021). On the other hand, low brain complexity can be found in the absence of slow waves in the ongoing brain activity even when measured at a finer spatial scale. For example, pharmacologically increasing excitation without blocking bistability results in wake-like spectral features associated with low perturbational complexity assessed by means of electrical stimulation in cortical slices (D’Andola *et al.* 2018). This finding can be understood if one considers that bistability, due to its intrinsic activity-dependent nature, is a latent neuronal property that prevents the emergence of large-scale cause-effect chains supporting brain complexity even when slow waves are not present in the ongoing prestimulus activity.

Another candidate postsynaptic mechanism that may account for the disruption of brain complexity is the decoupling between the apical and basal dendritic compartment of Layer 5 pyramidal neurons (Takahashi *et al.* 2016; Aru *et al.* 2020). Experiments in rodents have shown that this gating occurs under different anesthetics and that it may be controlled by higher-order thalamic nuclei (Suzuki and Larkum 2020; Takahashi *et al.* 2020). Crucially, the apical compartment receives feedbacks from corticocortical and thalamocortical loops, whereas the basal compartment mainly receives the feedforward stream from specific areas lower in the processing hierarchy. This dendritic decoupling is, at least in principle, in a key position to break down recurrent interactions across distributed areas, thus impairing functional integration in the brain. To the extent that this effect can be demonstrated at the whole-brain level and generalized to other conditions (e.g. sleep and coma), it may point to a fundamental neuronal determinant of the changes in complexity that are observed upon loss and recovery of consciousness. It will be important for future studies to explore whether and how neuronal decoupling and bistability, which are nonmutually exclusive, interact during physiological, pharmacological, and pathological loss of consciousness.

The examples discussed above suggest that we may soon develop a multiscale understanding, from single neurons to global brain measures, of the mechanisms of loss and recovery of brain complexity during different states of consciousness. Such optimistic predictions are justified for at least two reasons. The first reason is the availability of animal models in which clinically relevant complexity indices, such as PCI (Arena *et al.* 2021; Dasilva *et al.* 2021; Barbero-Castillo *et al.* 2021) or the repertoire of fMRI dynamics (Barttfeld *et al.* 2015), can already be measured and manipulated. Lesioning and optogenetic interventions in these models may e.g. reveal whether there are specific groups of neurons that are more important than others in sustaining complexity and whether we can directly act upon these nodes. Importantly, animal models grant direct access to subcortical structures that may play a key role, such as the thalamus (Redinbaugh *et al.* 2020). The second reason for optimism is the parallel development of whole-brain, data-driven computer simulations in which changes in complexity across different brain states are explicitly modeled at multiple scales (Deco *et al.* 2015; Zamora-López *et al.* 2016; Goldman *et al.* 2019). This computational neuroscience approach aims at offering a mechanistic framework for characterizing brain states in terms of the underlying causal mechanisms and the resulting complexity. Although discussing the role of computer simulations is clearly beyond the scope of the present paper, it is perhaps important to highlight that this kind of modeling effort, specifically pursued within the Human Brain Project, also aims at predicting the effects of pharmacological and electromagnetic perturbations needed to force transitions between brain states (Zamora-López *et al.* 2016; Kringelbach and Deco 2020). In a long-term perspective, employing this multiscale approach encompassing animal and *in silico* models holds the promise that we might learn, one day, how to act on cellular targets in order to restore complexity and possibly consciousness in injured human brains.

### Spatiotemporal scales of complexity

In humans, animal models, and computer simulations, complexity can be assessed at different spatiotemporal scales. As we have seen in the taxonomy section, neuroimaging studies of functional or effective connectivity in the brain examine interactions at the spatial level of voxels and at the temporal level of blood-oxygen fluctuations on the order of seconds. On the other hand, EEG-based measures of complexity explore a coarser spatial scale, but with much finer temporal sampling, on the order of milliseconds. These differences are expected to dramatically affect the estimation of brain complexity. For example, considering cortical areas at the time scale of seconds would necessarily minimize the estimates of differentiation by reducing the repertoire of possible states. At the other end of the spectrum, recordings of single neurons on a time window of a few milliseconds are likely to underestimate causal interactions, and thus integration, due to synaptic failures (Galarreta and Hestrin 1998), noise, and conduction delays (hundreds of milliseconds) characterizing large-scale communication. Where is the tradeoff? What is the optimal spatiotemporal grain at which measurements should be performed? Although recent advances in the mathematical analysis of multiscale systems have made progress in addressing the problem (Hoel *et al.* 2013, 2016; Rosas *et al.* 2020), determining in a principled way what counts as the “most appropriate” grain is of course very challenging.

Following the same logic that led to the identification of complexity as a relevant physical brain property, phenomenology may also guide the search for the optimal spatiotemporal scale. In this perspective, the appropriate grain at which complexity should be measured is the same spatiotemporal grain that is relevant for experience. For example, regarding time, there is a general agreement that an “instant” of experience is on the order of tens to hundreds of milliseconds rather than a few milliseconds or tens of seconds (Libet *et al.* 1991; Bachmann 2000; Holcombe 2009). Regarding space, experiments employing direct cortical stimulation in humans suggest that the minimal activated volume required to elicit phosphenes of different colors roughly corresponds to the size of a cortical minicolumn (Schmidt *et al.* 1996), whereas such color discrimination is lost when the activated volume is increased to the size of an hypercolumn (Tehovnik and Slocum 2007). Whether single neuron stimulation further increases perceptual differentiation or rather results in no perceptual effects at all is an important open question to be addressed in order to define the spatial grain at which neuronal interactions become relevant for consciousness. For the moment, taking into account minicolumns on a time window of hundreds of milliseconds may represent a reasonable starting point for measuring integration and differentiation in the brain. If we could work at such a scale, absolute complexity values would certainly skyrocket with respect to current readouts, making some justice to the actual complexity of the brain and allowing for more meaningful comparisons across experimental models, perhaps even between human and animal experiments.

### Localization of complexity

Another key question is whether the relevant complexity is generated in specific parts of the brain. It is indeed very likely that while some structural and functional arrangements are well suited for optimizing the coexistence of integration and differentiation, others may not. An extreme example is offered by the cerebellum. In this structure, myriads of microzones process inputs and produce outputs that are only feedforward (with no excitatory reverberant activity) and largely independent. In spite of the richness of the inputs (vestibular, visual, somatosensory, auditory, motor etc.) that various modules receive and process at an extraordinary pace, the strictly modular structure of the cerebellum is inherently incompatible with integration and high levels of complexity. In essence, considering its internal architecture, it is very likely that the cerebellum is not a single entity capable of high complexity but just an aggregate of small independent modules each traversed by a different data stream. This may explain why lesions of the cerebellum, which has four times more neurons than the cerebral cortex (Herculano-Houzel 2012), do not seem to affect consciousness (Lemon and Edgley 2010). Future imaging and electrophysiological studies are warranted to empirically confirm the difference between cerebellar and neocortical circuits with respect to their capacity to generate the relevant complexity.

Whether significant differences in the capacity for complexity can also be found within the cerebral cortex is an open question. Indeed, the cytoarchitectonics and the intrinsic arrangement of connections varies largely also across cortical areas. Especially in the posterior cortex, connections within each area are organized in a grid-like manner and across areas in a pyramid-like, convergent-divergent manner (Salin and Bullier 1995). This kind of architecture leads to a high level of systematic overlap in the connections among neurons, giving rise to a core network that “hangs together” tightly—in other words, it is functionally highly integrated (Haun and Tononi 2019; Deco *et al.* 2021). By contrast, cortical areas in which neurons are organized into more segregated modules (akin to the cerebellum), or in which the connections are organized more randomly, with less overlap, may be much less integrated. Available empirical evidence from macroscopic EEG and fMRI recordings in humans and monkeys suggest that posterior cortical areas are more relevant than others. Overall, these studies performed in sleep, propofol anesthesia (Luppi *et al.* 2019; Hahn *et al.* 2021) and post-comatose patients (King *et al.* 2013; Sitt *et al.* 2014; Luppi *et al.* 2019) converge in showing that loss and recovery of consciousness corresponds to changes in complexity located in posterior cortical regions.

The question of whether these regions are privileged by virtue of their particular grid-like structure (characterized by high density of connections, strong local connectivity, patchiness in the connectivity, and large numbers of short reentrant circuits) has also theoretical relevance. This in view of the recent debate highlighting the apparent paradox that grid-like structures, such as artificial expander graphs, that are easy to build due to their low algorithmic structural complexity, can give rise to high levels of integrated information (Aaronson 2014). The fact that similar structures can be found within the human brain and that they are more represented in some regions than in others, offers a unique opportunity to tackle this problem empirically. Arguably, starting from us humans, in whom phenomenology and physics can be compared directly, represents a first, mandatory step to understand the relationship between circuit architecture, the relevant complexity, and consciousness. For example, testing experimentally in humans whether neural circuits connected in a grid-like manner are hot spots for complexity and whether they are fundamental for sustaining the presence of consciousness and account for phenomenal properties, such as the experience of extended space (Haun and Tononi 2019), would provide valuable data to inform theoretical debates.

Given the likelihood that complexity is generated in some part of the brain but not in others, an additional problem will be defining the borders of the relevant subset of elements and whether these boundaries can shift in physiological or pathological states of consciousness. The relevance of this problem was already clear in the original formulation of the DCH, which defined a strategy to identify, based on statistical dependence (see above), the functional cluster (Tononi *et al.* 1998) generating the relevant complexity. This has been further qualified by a causal perspective in the latest formulation of Integrated Information Theory (IIT) (Oizumi *et al.* 2014). In a nutshell, the problem boils down to finding the borders that include the subset of elements that generate more complexity than any other, smaller or larger, subset. Although we can safely assume that some structures such as the retina and the cerebellum with their intrinsic lack of integration would rest outside of these borders, in practice, it will be very difficult to identify sharp edges within the brain. In fact, the possibility that these may even cut across cortical layers or neuronal populations makes an exhaustive search a daunting proposition.

### Dynamic and causal complexity

As already noted above, the example of the cerebellum is noteworthy as it suggests that some brain structures that are well suited for supporting important functions, and from which we can decode neural activity relevant to behavior (Jiang *et al.* 2015a; Friston and Herreros 2016), may not be necessary for consciousness because they do not have the appropriate internal causal structure. Although, to an external observer, the dynamics of cerebellar activity may seem complex, a causal approach by perturbing single microzones and by recording from the rest of the cerebellum is expected to reveal minimal interactions across modules and minimal complexity. In general, the possibility of a dissociation between complexity measures derived from the observation of ongoing brain dynamics and those based on the assessment of the underlying causal structure has interesting implications. Indeed, besides the intuitable case of the cerebellum, one can also conceive and test cases in which the opposite dissociation might occur, i.e. low dynamical complexity in ongoing activity with a concomitant high complexity of the underlying causal structure. For example, while measures of ongoing complexity tend to decrease when conscious subjects close their eyes and alpha rhythm becomes dominant (Stam *et al.* 1993), the perturbational complexity index does not change and remains high whether eyes are opened or closed (Casali *et al.* 2013). Perhaps more interestingly, one could even consider experiments investigating states of “pure consciousness” (Sullivan 1995), also known as “naked awareness” or “pure presence.” These conditions, whose phenomenology is well described by the century-old tradition of meditation practices, are characterized by a vividly present awareness yet devoid of any perceptual object and occur without monitoring the environment or self or attending to any particular content, as well as in the absence of reasoning and behaviors. How would these states, in which consciousness is vividly present while little function is performed and little information is being processed look like in terms of complexity measures? The limited literature currently available during meditation provide evidence of a reduction of complexity in the stream of observable ongoing brain dynamics (Aftanas and Golocheikine 2002; Irrmischer *et al.* 2018; Escrichs *et al.* 2019), whereas perturbation measures with TMS-EEG suggest the presence of a preserved underlying causal structure (Bodart *et al.* 2018a). Future studies, systematically performing these kinds of comparisons within the same individual will be very important to further define the practical implications inherent to the different approaches (e.g. observational vs perturbational) highlighted in the taxonomy section.

## Conclusion

To conclude, we would like to suggest how the body of convergent results highlighted by the present review may easily coalesce in a front where the field has a tangible opportunity to advance. For example, refining methods for measuring complexity, comparing their accuracy, and identifying their neuronal determinants at the appropriate spatiotemporal scale are practical steps within reach. These will be key to improve the way we assess consciousness and to gain the mechanistic insight needed to promote its recovery in pathological conditions.

Besides practical implications, connecting the dots of an interesting trajectory spanning a few decades of consciousness research bears conceptual relevance. In this respect, the early principles linking consciousness to complexity (Tononi and Edelman 1998) not only did provide a useful reference to provisionally taxonomize current empirical metrics but may also represent an interesting basis for future exchanges among different theoretical frameworks. For example, besides the Integrated Information Theory (Tononi 2004), which represents the direct evolution of the DCH, other frameworks, such as the Global Neuronal Workspace, the Kolmogorov Complexity Theory of Consciousness, and the Free Energy Principle, have more recently embraced, albeit starting from different premises, an explicit complexity-related framework (Dehaene *et al.* 2014; Ruffini 2017; Friston *et al.* 2020). Hence, while the focus on functional integration and differentiation seems to now constitute a common ground, other elements discussed in the present review may offer a concrete opportunity to explore interesting differences.

In this vein, one may ask a few specific questions. How do the different frameworks operationally define the boundaries of the subset of neurons generating the relevant complexity? The localization and extent of the physical substrate of consciousness is likely to differ depending on the way one answers this question. Also, why do some theories focus on the complexity of ongoing observable neuronal dynamics, whereas others emphasize the complexity of the underlying causal structure? It will be key to clarify this aspect, as it entails a substantially different understanding of the kind of information that matters for consciousness, extrinsic in the first case, intrinsic in the latter (Searle 2013; Koch and Tononi 2013). Finally, how do current theories consider the possibility that phenomenology-inspired principles related to complexity may be useful also in the search for content-specific neural correlates of consciousness? Asking this may sound premature now, but attempts have been made in this direction (Haun and Tononi 2019), and major initiatives, such as the Templeton Foundation’s Structured Adversarial Collaboration project, are in place to judge their merit.

Altogether, by considering the past positive trend highlighted in this review, we should all feel encouraged to face these kinds of questions head-on and to put forward specific principles and predictions, even if perceived as counterintuitive and hard to test at present. To the extent that these are both precise and daring, there are reasons to believe that they will likely be useful in 20 years from now.

## Supplementary Material

niab023_Supp

## Data Availability

There is no data associated with this manuscript.
