# Supplementary material for: Consciousness and complexity: a consilience of evidence
Source: Neurosci Conscious. 2021 Aug 30;2021(2):niab023. doi: 10.1093/nc/niab023 (PMC10941977; doi:10.1093/nc/niab023)
Supplement: niab023_Supp [file niab023_supp.zip › Suppl_Table.docx]

| **Year** | **First Author** | **Journal Abbreviation** | **DOI** | **Estimating Repertoire** | **Assessing Integration** |
| --- | --- | --- | --- | --- | --- |
| 2009 | Cauda F | J Neurol Neurosurg Psychiatry | 10.1136/jnnp.2007.142349 | 1 | O |
| 2009 | Vaudano AE | PLoS One | 10.1371/journal.pone.0006475 | 1 | O |
| 2010 | Pollonini L | Brain Topogr | 10.1007/s10548-010-0139-9 | 1 | O |
| 2010 | Spoormaker VI | J Neurosci | 10.1523/JNEUROSCI.2015-10.2010 | 1 | O |
| 2011 | Ku S-W | PLoS One | 10.1371/journal.pone.0025155 | 1 | O |
| 2011 | Lee U | Anesthesiology | 10.1097/ALN.0b013e31821102c9 | 1 | O |
| 2011 | Schrouff J | NeuroImage | 10.1016/j.neuroimage.2011.04.020 | 1 | O |
| 2011 | Song M | PLoS One | 10.1371/journal.pone.0017294 | 1 | O |
| 2011 | Zhou J | Conscious Cogn | 10.1016/j.concog.2010.08.003 | 1 | O |
| 2012 | Achard S | Proc Natl Acad Sci U S A | 10.1073/pnas.1208933109 | 1 | O |
| 2012 | Barrett AB | PLoS One | 10.1371/journal.pone.0029072 | 1 | O |
| 2012 | Boly M | Proc Natl Acad Sci U S A | 10.1073/pnas.1111133109 | 1 | O |
| 2012 | Nicolaou N | PLoS One | 10.1371/journal.pone.0033869 | 1 | O |
| 2012 | Schröter MS | J Neurosci | 10.1523/JNEUROSCI.6046-11.2012 | 1 | O |
| 2013 | Gili T | J Neurosci | 10.1523/JNEUROSCI.3480-12.2013 | 1 | O |
| 2013 | Guldenmund P | Brain Connect | 10.1089/brain.2012.0117 | 1 | O |
| 2013 | Kuhlmann L | PLoS One | 10.1371/journal.pone.0056434 | 1 | O |
| 2013 | Lee H | Anesthesiology | 10.1097/ALN.0b013e3182a8ec8c | 1 | O |
| 2013 | Lee U | Anesthesiology | 10.1097/ALN.0b013e31829103f5 | 1 | O |
| 2013 | Mäki-Marttunen V | Front Neuroinform | 10.3389/fninf.2013.00024 | 1 | O |
| 2013 | Monti MM | PLoS Comput Biol | 10.1371/journal.pcbi.1003271 | 1 | O |
| 2014 | Chennu S | PLoS Comput Biol | 10.1371/journal.pcbi.1003887 | 1 | O |
| 2014 | Crone JS | Neuroimage Clin | 10.1016/j.nicl.2013.12.005 | 1 | O |
| 2014 | Liu X | PLoS One | 10.1371/journal.pone.0092182 | 1 | O |
| 2014 | Maksimow A | PLoS One | 10.1371/journal.pone.0113616 | 1 | O |
| 2014 | Uehara T | Cereb Cortex | 10.1093/cercor/bht004 | 1 | O |
| 2014 | Untergehrer G | PLoS One | 10.1371/journal.pone.0087498 | 1 | O |
| 2014 | Varotto G | Clin Neurophysiol | 10.1016/j.clinph.2013.06.016 | 1 | O |
| 2015 | Crone JS | Neuroimage | 10.1016/j.neuroimage.2015.01.037 | 1 | O |
| 2015 | Lebedev AV | Hum Brain Mapp | 10.1002/hbm.22833 | 1 | O |
| 2015 | Li Q | J Neurol Sci | 10.1016/j.jns.2015.04.054 | 1 | O |
| 2015 | Liang P | Hum Brain Mapp | https://doi.org/10.1002/hbm.22914 | 1 | O |
| 2015 | Moon J-Y | PLoS Comput Biol | 10.1371/journal.pcbi.1004225 | 1 | O |
| 2015 | Palhano-Fontes F | PLoS One | 10.1371/journal.pone.0118143 | 1 | O |
| 2016 | Guevara Erra R | Phys Rev E | 10.1103/PhysRevE.94.052402 | 1 | O |
| 2016 | Kuceyeski A | Neuroimage Clin | 10.1016/j.nicl.2016.04.006 | 1 | O |
| 2016 | Liang Z | J Clin Monit Comput | 10.1007/s10877-015-9738-z | 1 | O |
| 2016 | Naro A | Brain Topogr | 10.1007/s10548-016-0489-z | 1 | O |
| 2016 | Tagliazucchi E | Brain Struct Funct | 10.1007/s00429-015-1162-0 | 1 | O |
| 2017 | Amico E | Neuroimage | 10.1016/j.neuroimage.2017.01.020 | 1 | O |
| 2017 | Blain-Moraes S | Front Hum Neurosci | 10.3389/fnhum.2017.00328 | 1 | O |
| 2017 | Chennu S | Brain | 10.1093/brain/awx163 | 1 | O |
| 2017 | Crone JS | Cereb Cortex | 10.1093/cercor/bhw112 | 1 | O |
| 2017 | Hashmi JA | Anesthesiology | 10.1097/ALN.0000000000001509 | 1 | O |
| 2017 | Lee M | Sci Rep | 10.1038/s41598-017-15082-5 | 1 | O |
| 2017 | Lioi G | Physiol Meas | 10.1088/1361-6579/aa81b5 | 1 | O |
| 2017 | Naro A | Neuroscience | 10.1016/j.neuroscience.2017.02.053 | 1 | O |
| 2017 | Viol A | Sci Rep | 10.1038/s41598-017-06854-0 | 1 | O |
| 2018 | Dell'Italia J | Front Neurol | 10.3389/fneur.2018.00439 | 1 | O |
| 2018 | Huang Y | Br J Anaesth | 10.1016/j.bja.2018.04.031 | 1 | O |
| 2018 | Li Y | PLoS One | 10.1371/journal.pone.0192358 | 1 | O |
| 2018 | Piarulli A | Sci Rep | 10.1038/s41598-018-24924-9 | 1 | O |
| 2018 | Sinitsyn DO | Hum Brain Mapp | 10.1002/hbm.24050 | 1 | O |
| 2019 | Afshani F | Cogn Neurodyn | 10.1007/s11571-019-09553-w | 1 | O |
| 2019 | Cacciola A | J Clin Med | 10.3390/jcm8030306 | 1 | O |
| 2019 | Lee H | Neuroimage | 10.1016/j.neuroimage.2018.12.011 | 1 | O |
| 2019 | Malagurski B | Neuroimage | 10.1016/j.neuroimage.2019.03.012 | 1 | O |
| 2019 | Pappas I | Anesthesiology | 10.1097/ALN.0000000000002977 | 1 | O |
| 2019 | Pappas I | Neuroimage | 10.1016/j.neuroimage.2018.10.078 | 1 | O |
| 2019 | Preller KH | Proc Natl Acad Sci U S A | 10.1073/pnas.1815129116 | 1 | O |
| 2019 | Rizkallah J | NeuroImage Clin | 10.1016/j.nicl.2019.101841 | 1 | O |
| 2019 | Viol A | Entropy | 10.3390/e21020128 | 1 | O |
| 2020 | Abeyasinghe PM | J Clin Med | 10.3390/jcm9051342 | 1 | O |
| 2020 | Barnett L | Neuroimage | 10.1016/j.neuroimage.2019.116462 | 1 | O |
| 2020 | Lee J-M | Sci Rep | 10.1038/s41598-020-59264-0 | 1 | O |
| 2020 | Nadin D | Neurosci Conscious | 10.1093/nc/niaa017 | 1 | O |
| 2020 | Pullon RM | Anesthesiology | 10.1097/ALN.0000000000003398 | 1 | O |
| 2020 | Rudas J | Brain Connect | 10.1089/brain.2019.0716 | 1 | O |
| 2020 | Wang S | Neuroimage Clin | 10.1016/j.nicl.2020.102188 | 1 | O |
| 2020 | Yan F | Clin EEG Neurosci | 10.1177/1550059420976303 | 1 | O |
| 2020 | Zhang R | Front Hum Neurosci | 10.3389/fnhum.2020.560586 | 1 | O |
| 2021 | Naro A | Int J Neural Syst | 10.1142/S0129065720500525 | 1 | O |
| 2019 | Lee M | Sci Rep | 10.1038/s41598-019-41274-2 | 1 | P |
| 2019 | Usami K | Sleep | 10.1093/sleep/zsz050 | 1 | P |
| 2005 | Burioka N | Clin EEG Neurosci | 10.1177/155005940503600106 | 2 | O |
| 2008 | Jordan D | Anesthesiology | 10.1097/ALN.0b013e31818d6c55 | 2 | O |
| 2009 | Lee U | Conscious Cogn | 10.1016/j.concog.2008.10.005 | 2 | O |
| 2010 | Li D | J Neural Eng | 10.1088/1741-2560/7/4/046010 | 2 | O |
| 2010 | Sarà M | Nonlinear Dynamics Psychol Life Sci | PMID: 20021774 | 2 | O |
| 2011 | Gosseries O | Funct Neurol | PMID: 21693085 | 2 | O |
| 2011 | Kaskinoro K | Br J Anaesth | 10.1093/bja/aer196 | 2 | O |
| 2011 | Sarà M | Neurorehab Neural Rep | 10.1177/1545968310378508 | 2 | O |
| 2011 | Wu D-Y | Clin Neurophysiol | 10.1016/j.clinph.2010.05.036 | 2 | O |
| 2013 | King J-R | Curr Biol | 10.1016/j.cub.2013.07.075 | 2 | O |
| 2013 | Lee GMH | Front Neuroinform | 10.3389/fninf.2013.00033 | 2 | O |
| 2013 | Tagliazucchi E | Proc Natl Acad Sci U S A | 10.1073/pnas.1312848110 | 2 | O |
| 2013 | Zorick T | PLoS One | 10.1371/journal.pone.0068360 | 2 | O |
| 2014 | Marinazzo D | Clin EEG Neurosci | 10.1177/1550059413510703 | 2 | O |
| 2015 | Liang Z | Clin Neurophysiol | 10.1016/j.clinph.2014.05.012 | 2 | O |
| 2015 | Schartner M | PLoS One | 10.1371/journal.pone.0133532 | 2 | O |
| 2016 | Andrillon T | J Neurosci | 10.1523/JNEUROSCI.0902-16.2016 | 2 | O |
| 2016 | Panda R | Front Hum Neurosci | 10.3389/fnhum.2016.00372 | 2 | O |
| 2016 | Piarulli A | J Neurol | 10.1007/s00415-016-8196-y | 2 | O |
| 2017 | Schartner MM | Neurosci Conscious | 10.1093/nc/niw022 | 2 | O |
| 2017 | Schartner MM | Sci Rep | 10.1038/srep46421 | 2 | O |
| 2017 | Wang J | Neurosci Lett | 10.1016/j.neulet.2017.05.045 | 2 | O |
| 2017 | Wislowska M | Sci Rep | 10.1038/s41598-017-00323-4 | 2 | O |
| 2018 | Eagleman SL | Front Neurosci | 10.3389/fnins.2018.00645 | 2 | O |
| 2018 | Isler JR | PLoS One | 10.1371/journal.pone.0206237 | 2 | O |
| 2018 | Mateos DM | Cogn Neurodyn | 10.1007/s11571-017-9459-8 | 2 | O |
| 2018 | Wielek T | PLoS One | 10.1371/journal.pone.0190458 | 2 | O |
| 2019 | Demertzi A | Sci Adv | 10.1126/sciadv.aat7603 | 2 | O |
| 2019 | Eagleman SL | PLoS One | 10.1371/journal.pone.0223921 | 2 | O |
| 2019 | Kim H | Entropy | 10.3390/e21100981 | 2 | O |
| 2019 | Lange N | Sci Rep | 10.1038/s41598-019-52949-1 | 2 | O |
| 2019 | Li D | Neuroimage | 10.1016/j.neuroimage.2019.03.076 | 2 | O |
| 2019 | Liu X | Brain Imaging Behav | 10.1007/s11682-018-9886-0 | 2 | O |
| 2019 | Miskovic V | Hum Brain Mapp | 10.1002/hbm.24393 | 2 | O |
| 2019 | Moser J | Front Syst Neurosci | 10.3389/fnsys.2019.00023 | 2 | O |
| 2019 | Wenzel M | Cell Syst | 10.1016/j.cels.2019.03.007 | 2 | O |
| 2020 | Frohlich J | Neurosci Conscious | 10.1093/nc/niaa005 | 2 | O |
| 2020 | Hou F | Sleep | 10.1093/sleep/zsaa226 | 2 | O |
| 2020 | Liang Z | IEEE Trans Neural Syst Rehabil Eng | 10.1109/TNSRE.2020.2964819 | 2 | O |
| 2020 | Martens G | Neuroimage Clin | 10.1016/j.nicl.2020.102426 | 2 | O |
| 2020 | Wang Y | Int J Neurosci | 10.1080/00207454.2019.1702543 | 2 | O |
| 2020 | Wu S-J | Entropy | 10.3390/e22121411 | 2 | O |
| 2019 | Colombo MA | Neuroimage | 10.1016/j.neuroimage.2019.01.024 | 2 | O+P |
| 2020 | Farnes N | PLoS One | 10.1371/journal.pone.0242056 | 2 | O+P |
| 2005 | Massimini M | Science | 10.1126/science.1117256 | 2 | P |
| 2010 | Ferrarelli F | Proc Natl Acad Sci USA | 10.1073/pnas.0913008107 | 2 | P |
| 2010 | Massimini M | Cogn Neurosci | 10.1080/17588921003731578 | 2 | P |
| 2012 | Rosanova M | Brain | 10.1093/brain/awr340 | 2 | P |
| 2013 | Casali AG | Sci Transl Med | 10.1126/scitranslmed.3006294 | 2 | P |
| 2013 | Ragazzoni A | PLoS One | 10.1371/journal.pone.0057069 | 2 | P |
| 2015 | Pigorini A | NeuroImage | 10.1016/j.neuroimage.2015.02.056 | 2 | P |
| 2015 | Sarasso S | Curr Biol | 10.1016/j.cub.2015.10.014 | 2 | P |
| 2015 | Usami K | Hum Brain Mapp | 10.1002/hbm.22948 | 2 | P |
| 2016 | Bai Y | Front Neurosci | 10.3389/fnins.2016.00473 | 2 | P |
| 2016 | Casarotto S | Ann Neurol | 10.1002/ana.24779 | 2 | P |
| 2017 | Bodart O | Neuroimage Clin | 10.1016/j.nicl.2017.02.002 | 2 | P |
| 2018 | Bodart O | Brain Stimul | 10.1016/j.brs.2017.11.006 | 2 | P |
| 2018 | Rosanova M | Nat Commun | 10.1038/s41467-018-06871-1 | 2 | P |
| 2019 | Comolatti R | Brain Stimul | 10.1016/j.brs.2019.05.013 | 2 | P |
| 2019 | Ruiz de Miras J | Comput Methods Programs Biomed | 10.1016/j.cmpb.2019.04.017 | 2 | P |
| 2020 | Lutkenhoff ES | Brain Stimul | 10.1016/j.brs.2020.07.012 | 2 | P |
| 2020 | Sinitsyn DO | Brain Sci | 10.3390/brainsci10120917 | 2 | P |
| 2010 | Lee U | Anesthesiology | 10.1097/ALN.0b013e3181f229b5 | 3 | O |
| 2012 | Fingelkurts AA | Open Neuroimag J | 10.2174/1874440001206010055 | 3 | O |
| 2013 | Fingelkurts AA | Clin EEG Neurosci | 10.1177/1550059412474929 | 3 | O |
| 2013 | Shin J | PLoS One | 10.1371/journal.pone.0070899 | 3 | O |
| 2014 | Alonso LM | Front Neural Circuits | 10.3389/fncir.2014.00020 | 3 | O |
| 2015 | Allegrini P | Phys Rev E | 10.1103/PhysRevE.92.032808 | 3 | O |
| 2016 | Fingelkurts AA | Open Neuroimag J | 10.2174/1874440001610010041 | 3 | O |
| 2017 | Fingelkurts AA | Clin EEG Neurosci | 10.1177/1550059417696180 | 3 | O |
| 2017 | Mateos DM | Phys Rev E | 10.1103/PhysRevE.96.062410 | 3 | O |
| 2018 | Cavaliere C | Front Neurol | 10.3389/fneur.2018.00861 | 3 | O |
| 2018 | Kim H | PLoS Comput Biol | 10.1371/journal.pcbi.1006424 | 3 | O |
| 2019 | Bocaccio H | J R Soc Interface | 10.1098/rsif.2019.0262 | 3 | O |
| 2019 | Escrichs A | Front Syst Neurosci | 10.3389/fnsys.2019.00027 | 3 | O |
| 2019 | Kung Y-C | Hum Brain Mapp | 10.1002/hbm.24590 | 3 | O |
| 2020 | Cai L | J Neural Eng | 10.1088/1741-2552/ab8b2c | 3 | O |
| 2020 | Dürschmid S | PLoS One | 10.1371/journal.pone.0233589 | 3 | O |
| 2013 | Jordan D | Anesthesiology | 10.1097/ALN.0b013e3182a7ca92 | 1+2 | O |
| 2016 | Claassen J | Ann Neurol | 10.1002/ana.24752 | 1+2 | O |
| 2016 | Huang Z | Neuroimage | 10.1016/j.neuroimage.2015.08.062 | 1+2 | O |
| 2017 | Lee H | Hum Brain Mapp | 10.1002/hbm.23708 | 1+2 | O |
| 2018 | Kim H | Front Hum Neurosci | 10.3389/fnhum.2018.00042 | 1+2 | O |
| 2018 | Lee H | Entropy | 10.3390/e20070518 | 1+2 | O |
| 2018 | Stefan S | Brain Topogr | 10.1007/s10548-018-0643-x | 1+2 | O |
| 2019 | Imperatori LS | Sci Rep | 10.1038/s41598-019-45289-7 | 1+2 | O |
| 2019 | Lioi G | Anaesthesia | https://doi.org/10.1111/anae.14535 | 1+2 | O |
| 2020 | Carrière M | Brain Sci | 10.3390/brainsci10070469 | 1+2 | O |
| 2020 | Dheer P | Heliyon | 10.1016/j.heliyon.2020.e05769 | 1+2 | O |
| 2020 | Huang H | Neurocrit Care | 10.1007/s12028-020-01051-w | 1+2 | O |
| 2020 | Imperatori LS | Sleep | 10.1093/sleep/zsaa247 | 1+2 | O |
| 2020 | Liang Z | Anesthesiology | 10.1097/ALN.0000000000003015 | 1+2 | O |
| 2020 | Varley TF | Sci Rep | 10.1038/s41598-020-57695-3 | 1+2 | O |
| 2020 | Varley TF | PLoS One | 10.1371/journal.pone.0223812 | 1+2 | O |
| 2020 | Varley TF | Neuroimage | 10.1016/j.neuroimage.2020.117049 | 1+2 | O |
| 2019 | Xia X | Neuroreport | 10.1097/WNR.0000000000001362 | 1+2 | P |
| 2014 | Sitt JD | Brain | 10.1093/brain/awu141 | 1+2+3 | O |
| 2018 | Engemann DA | Brain | 10.1093/brain/awy251 | 1+2+3 | O |
| 2019 | Luppi A | Nat Commun | 10.1038/s41467-019-12658-9 | 1+2+3 | O |
| 2020 | Sangare A | Brain Sci | 10.3390/brainsci10110845 | 1+2+3 | O |
| 2012 | Chu CJ | J Neurosci | 10.1523/JNEUROSCI.5669-11.2012 | 1+3 | O |
| 2018 | Di Perri C | Hum Brain Mapp | 10.1002/hbm.23826 | 1+3 | O |
| 2019 | Golkowski D | Anesthesiology | 10.1097/ALN.0000000000002704 | 1+3 | O |
| 2020 | Cai L | J Neural Eng | 10.1088/1741-2552/ab79f5 | 1+3 | O |
| 2021 | Hahn G | Neuroimage | 10.1016/j.neuroimage.2020.117470 | 1+3 | O |
| 2021 | Luppi AI | Neuroimage | 10.1016/j.neuroimage.2020.117653 | 1+3 | O |
| 2014 | Tagliazucchi E | Hum Brain Mapp | 10.1002/hbm.22562 | 2+3 | O |
| 2016 | Tagliazucchi E | J R Soc Interface | 10.1098/rsif.2015.1027 | 2+3 | O |
